# Genomic Characterization of Three Canadian Mumps Outbreaks Demonstrates Endemic Transmission in Canada

**DOI:** 10.3390/v16081280

**Published:** 2024-08-10

**Authors:** Jasmine Rae Frost, Grace Eunchong Seo, Kerry Dust, Jared Bullard, Peter Daley, Jason J. LeBlanc, Joanne Hiebert, Elizabeth McLachlan, Alberto Severini

**Affiliations:** 1 Department of Medical Microbiology and Infectious Diseases, Faculty of Health Sciences, University of Manitoba, Winnipeg, MB R3E 0J9, Canada; jasmine.frost@phac-aspc.gc.ca (J.R.F.); grace.seo@phac-aspc.gc.ca (G.E.S.); jared.bullard@phac-aspc.gc.ca (J.B.); 2 Cadham Provincial Lab, Shared Health Manitoba, Winnipeg, MB R3E 3J7, Canada; kerry.dust@gov.mb.ca; 3 Discipline of Medicine and Laboratory Medicine, Memorial University, St. John’s, NL A1B 3V6, Canada; pkd336@mun.ca; 4 Division of Microbiology, Department of Pathology and Laboratory Medicine, Nova Scotia Health, Halifax, NS B3H 1V8, Canada; jason.leblanc@nshealth.ca; 5 Department of Pathology, Dalhousie University, Halifax, NS B3H 1V8, Canada; 6 National Microbiology Laboratory Branch, Public Health Agency of Canada, Winnipeg, MB R3E 3R2, Canada; joanne.hiebert@phac-aspc.gc.ca (J.H.); elizabeth.mclachlan@phac-aspc.gc.ca (E.M.)

**Keywords:** mumps virus, whole genome sequencing, Canadian mumps outbreaks

## Abstract

Despite the provision of a mumps vaccination program in Canada for over three decades, mumps has not reached elimination. Instead, a re-emergence has been observed in vaccinated populations, particularly in young adults. These outbreaks have been almost exclusively due to genotype G infections, a trend that has been seen in other countries with high mumps vaccination rates. To characterize mumps outbreaks in Canada, genomes from samples from Manitoba (*n* = 209), Newfoundland (*n* = 25), and Nova Scotia (*n* = 48) were sequenced and analysed by Bayesian inference. Whole genome sequencing was shown to be highly discriminatory for outbreak investigations compared to traditional Sanger sequencing. The results showed that mumps virus genotype G most likely circulated endemically in Canada and between Canada and the US. Overall, this Canadian outbreak data from different provinces and ancestral strains demonstrates the benefits of molecular genomic data to better characterize mumps outbreaks, but also suggests genomics could further our understanding of the reasons for potential immune escape of mumps genotype G and evolution in highly vaccinated populations. With a possible endemic circulation of mumps genotype G and the remaining risk of new imported cases, increased surveillance and alternative vaccination strategies may be required for Canada to reach the current target for mumps or a future elimination status.

## 1. Introduction

In Canada, the measles, mumps, and rubella (MMR) vaccine has been implemented in provincial childhood immunization since 1996/97 as a two-dose regimen which is the current recommendation for MMR use in Canada [1,2,3]. While the MMR vaccine contributed to achieving the elimination of measles and rubella in Canada, sporadic mumps cases and outbreaks continue to emerge [3,4]. Initially the MMR vaccine reduced mumps cases by 99%, but recent years have seen a resurgence in the number of mumps cases. Mumps resurgence is a worldwide trend with similar characteristics in regions with high vaccine coverage [5,6,7,8,9]. Instead of infections occurring in young children as they had prior to the introduction of the vaccine, these mumps outbreaks occur among vaccinated adults, and are caused almost exclusively by mumps virus genotype G strains [5,6,7,8,9].

From 2016 to 2018, 24 mumps outbreaks were reported in nine provinces in Canada, with a combined total of 881 cases [4]. Within these outbreaks, the most commonly infected age group was 15–39 years, and of those whose vaccination status was known, 49% were fully vaccinated [4]. In addition to these 24 outbreaks, a large multi-year outbreak occurred in Manitoba (MB) starting in the fall of 2016 with a vaccinated university student. This outbreak continued until the end of 2018, with a total of 2223 confirmed cases and 1566 cases (70.4%) of these were reported in the Northern Regional Health area [10]. Similar to mumps outbreaks seen in countries with high vaccine coverage, this outbreak was caused by genotype G strains [10]. The incidence rates were highest in those aged 18–29 years, and of those whose vaccination data were available, 70% of cases had at least two doses of an MMR vaccine [10]. These findings were consistent with findings of previous mumps outbreaks with genotype G in other Canadian provinces, and other countries with high vaccine coverage [4,11,12].

These outbreaks have demonstrated a lack of efficacy of mumps vaccination for mumps genotype G and raise questions about the success of mumps control in Canada. The National Advisory Committee for Immunization (NACI) has recommended the administration of a third MMR dose in outbreak situations [3]. While this has been used in university outbreak settings and may be helpful in outbreak control, it is still unknown whether a third dose will offer long-lasting protection [13,14]. Immune response to mumps virus likely involves many host factors, but with most recent mumps outbreaks being nearly all genotype G suggests possible viral mutations may have arisen that are able to escape MMR vaccine immunity.

Whole genome sequencing (WGS) may contribute valuable insight to inform public health response to mumps epidemics. A study of multiple Canadian outbreaks using WGS has not yet been conducted. Our study includes the large 2016–2018 outbreak in Manitoba, as well as concurrent mumps outbreaks in Newfoundland (NL) and Nova Scotia (NS), to expand geographical representation. Our objective was to describe the rate of mutation of mumps within these outbreaks, evaluate the endemicity of mumps, and identify potential host or viral factors that could explain the predominance of mumps genotype G infection.

## 2. Methods

### 2.1. Ethics

This study was approved by the Health Research Ethics Board (HREB) at the University of Manitoba [HS23597 (H2020:035)]. For NL, HREB approval was obtained to share epidemiological data for outbreak samples (#20190586, 2018.176). For NS, sequence-based characterization of the mumps virus is part of routine public health investigations and was exempt from HREB approval.

### 2.2. Sample Selection for Provincial Outbreaks

In MB, the Cadham Public Health Laboratory randomly selected 10% of mumps outbreak samples from each regional health authority for a total of 266 samples. Only samples with a PCR cycle threshold (Ct) value of <30.5 cycles were included to improve WGS success. In NL and NS, all mumps RT-PCR positive samples with sufficient volume spanning 2017–2018 were selected for WGS (36 and 62 specimens, respectively).

### 2.3. Demographic and Epidemiological Data Collection

Demographic and epidemiological data extraction was performed for the Manitoba outbreak by the Manitoba Center for Health Policy (MCHP) through the use of scrambled Personal Health Identification Number (PHIN) that was provided to MCHP by the Health Information Privacy Committee. Data were extracted from the Cadham Public Health Laboratory LIMS database, Manitoba Immunization Monitoring Data, and the Manitoba Health Insurance registry. Vaccination records, age, sex, postal code and income level were fields that were collected for each Manitoba sample included in the study. MCHP classifies urban Manitoba to postal codes that are within the cities of Winnipeg and Brandon, and all others are classified as rural. MCHP set income levels in 5 quartiles where 20% of the population falls into each. These are then divided into rural (R) and urban (U) settings. In the models used in this study, income was set to Low (quartiles 1 and 2) or High (quartiles 3 to 5), as shown in Table 1.

Newfoundland Public Health provided the following demographic and epidemiological information: age, sex, date of onset, community of residence, epidemiological linkage to a known case and vaccination status for a subset of samples. Only cases within the Eastern Health region were included.

Demographic and epidemiological data for the Nova Scotia samples were not divulged by Public Health.

### 2.4. SH Gene Sequencing

Most study samples were retrospective samples that had previously been genotyped by the National Microbiology Laboratory using Sanger sequencing of a 316 nucleotide region of the small hydrophobic (SH) gene [15]. Novel samples for this study that were provided by Cadham Provincial Laboratory were genotyped in the same manner [15].

### 2.5. Whole Genome Sequencing (WGS)

WGS was performed as previously described using the probe enrichment method for MuV [16]. Final libraries were sequenced on the MiSeq platform using a MiSeq Reagent Kit v3 (600-cycle, Cat #MS-102-3003) [16].

Sequence analysis was carried out on the Galaxy platform V20.01 [17] and the consensus genotype G mumps sequence constructed for the enrichment probe design was used as a reference for alignment [16]. FastQ files obtained from the MiSeq sequencing run were first trimmed using Trimmomatic v0.36.5 (settings: sliding window trimming, number of bases to average across: 4 and average quality required: 20) [18]. Samples were then aligned using Bowtie 2 v2.3.4.2 [19] with the setting “pair dataset collection” and a maximum fragment length of 1000. All other settings were left as default. The “.bam” files generated from the Bowtie alignment were used to view aligned sequences in the graphical interface program known as Tablet, to determine which samples had full coverage [20]. The definition of the full genome used for this project was contiguous sequences with at least 10x sequencing coverage from the start codon of the N gene to the stop codon of the L gene (the entire genome minus the non-coding termini (WGS-t)). The SNVPhyl pipeline v1.2.3 was run on the Galaxy pipeline with complete genome sequences, and was used to identify mutations within complete genomes [17,21]. Here the settings used are as follows: minimum coverage-3, minimum mean mapping-28, and a single nucleotide variations (SNV) abundance ratio of 0.75. In addition to this, under the step “Consolidate VCF’s” the option SNV density filtering was turned off [21].

Phylogenetic trees were constructed by maximum likelihood analysis using IQ-TREE v2.1.3 with the following settings: -m TEST (model testing), -b 1000 (bootstrap analysis), -keep-ident [22,23,24]. Additionally, likelihood mapping was carried out using IQ-TREE and the setting –lmap 5000 [25]. Trees were subsequently annotated using iTOL v6.8.2 [26]. These sequences are available on GenBank under the accession numbers PQ156981–PQ157298.

### 2.6. Bayesian Analysis of Genomic Sequences

BEAST 2 v1.10.4 was used for the Bayesian phylogenetics [27]. To determine the appropriate model, the constant size, exponential growth and the Bayesian skyline coalescent tree priors were tested using a strict molecular clock and a relaxed molecular clock [28,29,30,31,32,33]. BEAST runs were run in triplicate with a chain length of 50,000,000. Trees were sampled every 5000 chains (Supplementary Table S1).

Final BEAST parameters used the HYK substitution model with empirical frequencies, a clock rate of 4 × 10^−4^ mutations/site/year, strict molecular clock and the coalescent exponential clock with a normal clock rate [28,29,33]. Runs used 100,000,000 chains and sampled every 10,000. The final set was run in triplicate. Trees were annotated using the BEAST program Tree Annotator v2.7.6, and a 10% burn-in. The resulting trees were viewed using the program Fig Tree v1.4.4, which allowed for the ordering of nodes and colour coding of branches by province. To investigate the relationship between the three outbreaks, archival Canadian samples and international samples, additional samples were added to the final data set. The final sample set contained successful sequencing results for samples from MB (*n* = 209), NL (*n* = 25), and NS (*n* = 48). These were analysed along with archival Canadian samples (2 per year based on availability and SH gene profiles similar to the WHO Sheffield reference strain) from 2006–2020 (excluding 2017–2018); 5 Canadian BC samples that showed a different SH gene profile and therefore were not related to the 2017–2018 outbreak; and representative mumps genotype G whole genome international samples (accession numbers: MT880127.1, MT880125.1, MT238687.1, MT238686.1, MT238683.1, MT238681.1, MH638235.1, MH638234.1, MG986446.1, MG986432.1, MG986426.1, MG986417.1, MG986410.1, MG986395.1, MG986385.1, MG986381.1, MG765427.1, MG765426.1, MG460606.1, MF965318.1, MF965317.1, MF965313.1, MF965260.1, MF965258.1, MF965232.1, KY604739.2).

### 2.7. Mutation Analysis

An alignment of the National Microbiology Laboratory real-time mumps F-gene primers was done using Geneious Prime V 11.0.20.1 on the WGS-t data set. The primers analysed were Mps5618f (TCTCACCCATAGCAGGGAGTTATAT), Mps5696r (GTTAGACTTCGACAGTTTGCAACA) and the Taqman probe Mps5644p (6FAM-AGGCGATTTGTAGCACTGGATGGAACA-MGB).

Single nucleotide variations (SNVs) were identified by the SNVPhyl pipeline. 2341 SNVs were identified, and from these we selected those that occurred in at least 2% of samples (*n* = 430). Only non-synonymous substitutions (leading to a change in amino acid) were investigated further. Gene nucleotide sequences were translated via the site http://bio.biomedicine.gu.se/edu/translat.html (accessed on July 2022). The amino acid sequences were then compared to the consensus described above using the site https://web.expasy.org/sim/ (accessed on July 2022).

Mutation profile figures were created using SNVPhyl pipeline outputs, indexed with bcftools v1.10.2. Using R Studio v1.4.1717, data were arranged and plotted using ggplot. R packages used were tidyverse, grid, stringr, ggplot2, and ggpubr.

The mean relative evolutionary rate was determined for each nucleotide in the mumps virus WGS-t genome using MEGA 11 v11.0.13 [34,35]. The mean evolutionary rates are scaled so the average rate is 1 across the genome, meaning a rate above one indicates a faster evolution. The average mean relative evolutionary rate was taken for 100bp across the genome and plotted.

### 2.8. Statistical Analysis

SAS 9 (SAS Institute Inc. 2013, Cary, NC, USA) was used to perform risk factor analysis on the Manitoba samples, using a logistic regression model. Location, income and sex were analysed as categorical variables and age was analysed as a continuous variable.

## 3. Results

**Figure 1:**
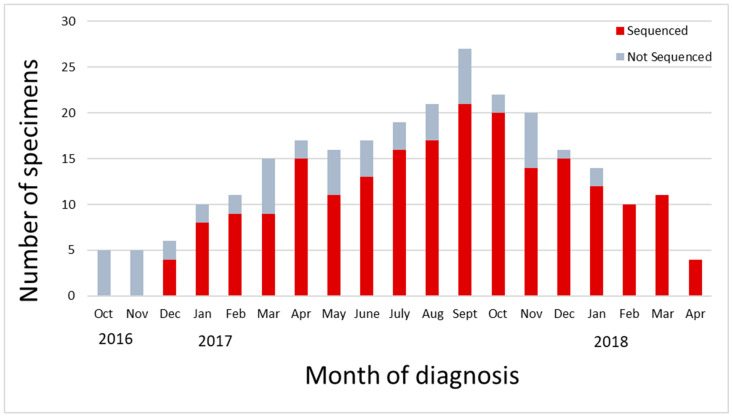
Distribution of samples selected in Manitoba for WGS-t sequencing spanning the 2016–2018 outbreak. Samples were chosen by provincial regional health authority; a total of 10% of total outbreak samples were included in this data set. Successfully sequenced samples are shown in red, unsuccessful samples in grey.

Of the available samples, successful sequences were obtained with 78.6% (209/266) of the MB samples, as well as 69.4% (25/36) NL samples and 77.4% (48/62) of samples from NS. These sequences had a minimum of 10x coverage for all nucleotide positions (excluding the non-coding termini). Figure 1 shows in red the number of samples that were successfully sequenced from the large Manitoba outbreak, and in grey, those that were unsuccessful.

For the 209 MB samples, each was categorized but receipt of MMR if data was available: 0 doses (*n* = 33), 1 dose (*n* = 40), 2 doses (*n* = 85), and 3 doses (*n* = 14). Of the 25 NL samples with successful sequences, 4 were known to have received one dose of MMR and 11 had two doses. Of the 209 MB samples with sequences, 124 and 48 individuals came from rural and urban areas, respectively. Female and male sex was nearly equally distributed at 91 and 81, respectively. When classification was based on income, 126 had low income whereas 43 had high income. Further stratification of income into quartiles and urban/rural were also determined (Table 1).

### 3.1. Comparison between Traditional SH Gene Sequencing and WGS

Previous SH gene sanger sequencing had shown that Canadian MuV had an SH gene profile similar to that of the WHO genotype G reference stain (Sheffield). Using the SH gene sequences derived from Sanger sequencing for samples from outbreaks in MB, NL, and NS, a maximum likelihood tree was generated (bootstrap analysis of 1000) [22,24]. As there is limited genetic diversity within the SH gene of these samples, this analysis resulted in very low bootstrap values, with many branches having a value of less than 50 (Supplementary Figure S1). This indicates that the traditional method of characterizing mumps virus using Sanger sequencing of the SH gene does not contain enough genetic diversity for fulsome phylogenetic analyses, or the outbreaks are related. WGS was explored to investigate whether the three outbreaks could be further discriminated.

**Figure 2:**
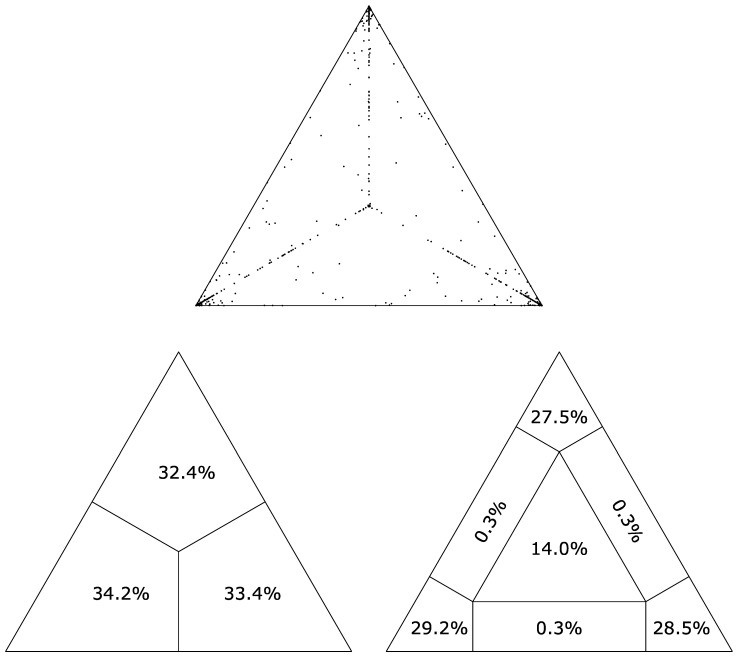
Likelihood mapping of MuV whole genome sequences (WGS-t) from the Manitoba, Newfoundland, and Nova Scotia outbreaks. IQ-TREE 2.1.3 was run with 5000 quartets indicated. The top triangle indicates the distribution of quartets. The left triangle indicates the percentage of quartets falling into one of the 3divided areas. Most importantly, the bottom right triangle shows how the samples were placed, with 85.2% being placed at the corners, indicating strong phylogenetic power.

From the WGS-t data obtained from the outbreak samples, a likelihood analysis was performed to test for phylogenetic power. The analysis of these samples in IQ-Tree with a quartet value of 5000 showed that 85.2% of sequences fell into the corners indicating they were fully resolved, and another 0.9% were at the sides where two topologies are equally possible Figure 2 [22,35]. This indicated that the data set was appropriate to use for further phylogenetic analysis, and the WGS data showed better discrimination than SH sequencing alone. In contrast, when a likelihood analysis was run on the SH gene dataset, only 45.2% of sequences fell into the corners, indicating resolution, and 54.8% were unable to be properly resolved (Supplementary Figure S2).

**Figure 3:**
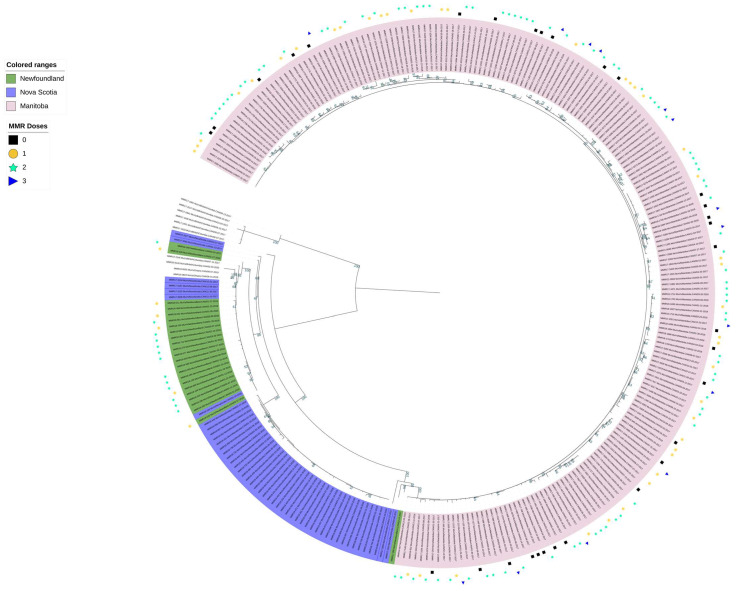
Maximum likelihood tree of mumps virus genome sequences. Illustrated are whole genomes from outbreaks in MB (pink), NL (green), and NS (blue). Samples from an outlier outbreak from BC was also included. This analysis was run using IQ-Tree with a bootstrap value of 1000 (bootstrap values are shown in teal to the left of the tree, those lower than 40 are not shown). Annotation of the tree was done using iTOL. The vaccination status (if known) is displayed on the outside of the tree. The tree can also be viewed at https://itol.embl.de/shared/18FQx6JYPbiX5.

This whole genome data set was also used to run a maximum likelihood tree with a bootstrap analysis of 1000. The WGS data set was also used to run a maximum likelihood tree with a bootstrap analysis of 1000. With the full WGS-t genomes, samples clustered based on province and the bootstrap results were higher, again showing that WGS provided a better phylogenetic resolution than SH gene sequencing (Figure 3). When MMR vaccination status was added to the tree (available for some MB and NL samples only), there was no obvious clustering based on vaccination cohorts having received 0, 1, 2, or 3 MMR doses (Figure 3).

### 3.2. Bayesian Analysis

BEAST was used to investigate phylogenetic patterns over time. A time-inferred tree was required to determine when the evolution of each provincial outbreak occurred and if they overlapped with archival sequences in Canada, indicating the possibility of endemic transmission of mumps in Canada. All MB, NL, and NS outbreak samples were analysed, along with two archival Canadian samples per year from 2006–2020 (excluding 2017–2018 as these years were already represented), five genotype G British Columbia (BC) samples that showed a different SH gene profile and unrelated to the 2017–2018 outbreak, and representative samples of publicly available genotype G whole genome sequences form international samples.

**Figure 4:**
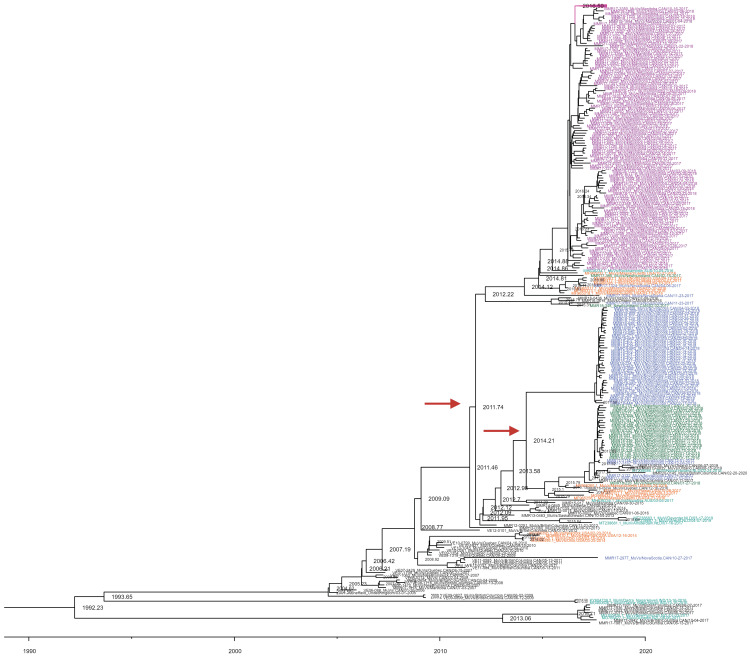
Whole genome sequencing (WGS-t) BEAST tree of mumps virus sequences. Samples included 209 from MB (purple), 25 from NL (green) and from 48 NS (blue), archival Canadian sequences (black) along with samples from the US (orange), international (teal). A portion of Manitoba samples have been collapsed to better view the image. Additionally, redundant branch labels have been removed to allow for readability. Red arrows indicate divergent dates between provincial outbreaks.

Samples that had closely related SH gene sequences (with 1 to 5 site differences) when compared to the MuVi/Sheffield.GBR/1.05 World Health Organization (WHO) reference strain for mumps virus genotype G showed a divergence date of 1993 in the whole genome Bayesian tree. The tree shows the MB outbreak diverged from NS and NL in 2011, and the latter two provinces diverged from each other in 2014 (Figure 4). While the outbreaks alone appear to be separate, the inclusion of archival Canadian sequences (shown in black) highlights continual evolution, which suggests endemic circulation of mumps in Canada. These archival sequences can be found both at the bottom of the tree and within the provincial outbreaks. The Bayesian analysis determined that the average clock rate for Canadian samples was 3.84 × 10^−4^ mutations/site/year (95% CI: 3.38 × 10^−4^–4.29 × 10^−4^).

Mumps WGS data from the USA (*n* = 16, from 6 different states, shown in orange) also cluster closely to these Canadian sequences, suggesting possible transmission and evolution between Canada and the USA. Other international sequences obtained from the Netherlands (*n* = 4) and one sequence from Australia also clustered within the Canadian outbreaks.

The British Columbia genotype G samples that were found to have a different SH gene profile through Sanger sequencing show a divergence of 1992 in the Bayesian analysis. The Bayesian tree shows a distinct branching of this clade, suggesting it is a distinct importation event. These samples cluster strongly with samples from India and New Zealand (Figure 4).

### 3.3. Mutation Data

The WGS-t data set allowed for an investigation into the current National Microbiology Laboratory real-time primers for the mumps F gene. Mapping the forward, and reverse primers and the probe to this data set revealed only one mutational difference. The Mps5644p probe had an A to G mutation as indicated by the bold letter (AGGCGATTTGTAGCACTGGATGGAACA). This mutation was present in 199 (70.57%) of samples.

**Figure 5:**
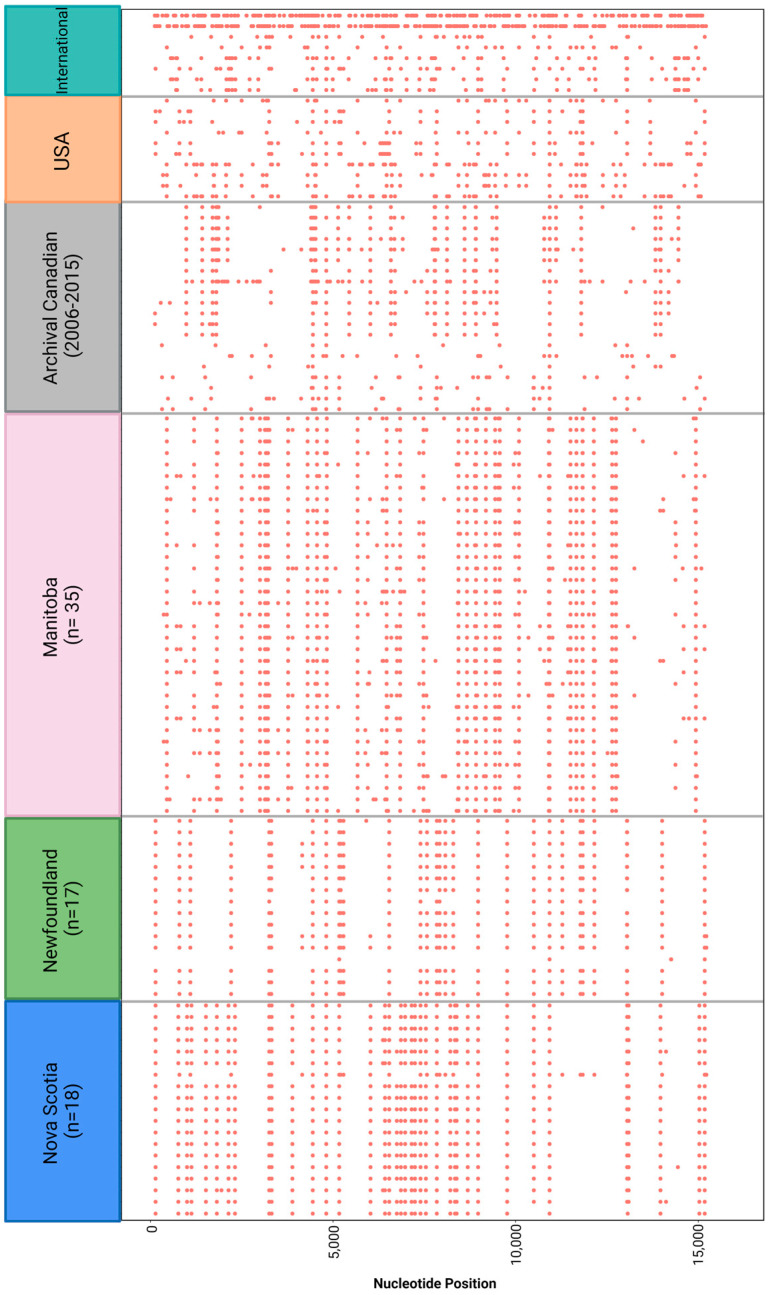
Mutational profile of successful mumps virus genome sequences. A representative sample of provincial sequences from 2018 were selected (ordered by date of receival at the NML), along with archival Canadian sequences with SH profiles similar to the WHO reference Sheffield strain, a selection of USA sequences: MF965313.1, MF965260.1, MF965258.1, MF965232.1, MT880127.1, MT880125.1, MG986426.1, MG986385.1, MG986417.1, MG986410.1 and international sequences representing countries that had publicly available genotype G whole genomes at the time of analysis: MG460606.1, MG765426.1, MH638235.1, MH638234.1, MT238687.1, MT238686.1, MT238683.1, MT238681.1. Nucleotide variations from the consensus are highlighted by the coloured dots. Groups are divided by a grey line.

When SNVs were explored from the outbreak specimen WGS data, 2341 SNVs were identified, but only those occurring in at least 2% of samples (*n* = 430) were pursued. Of these, only non-synonymous substitutions were investigated. Some were conserved across all three Canadian provinces (A1813T), while several other variants were province specific. For example, SNVs exclusive to MB included G2996A, C4828T, G5949T, A6334G, T6350C, T6353C, A7358A, and C8412T (Figure 5). In addition, the NL/NS outbreak shared SNVs A134G, G6019A, and A8072G, but NS also had unique SNVs (A1513G, C2312A, T3314A, G3882A, A6416C, C6744T).

**Figure 6:**
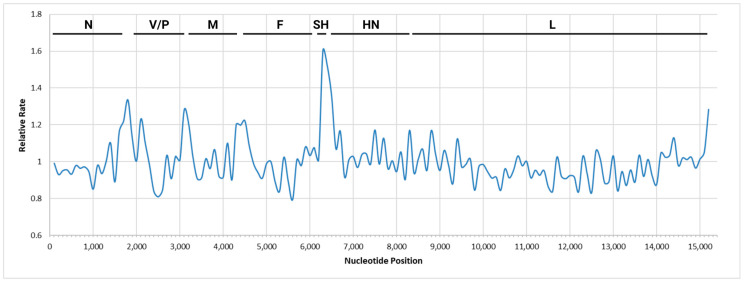
The mean relative evolutionary rate was determined for each nucleotide in the mumps virus WGS-t genome. Then the average mean relative evolutionary rate was taken for 100bp across the genome and this was plotted. Sites showing a rate lower than 1 are evolving slower than average, and those showing a rate above 1 are evolving faster than average. The analysis was run in MEGA-11 using the aligned dataset that included outbreak samples from MB, NL, and NS as well as archival outbreak samples as described above. Black bars at the top of the graph represent the coding region for each mumps virus gene.

It was of interest to determine if there was a region of the mumps viral genome other than the currently used SH gene that contained a high level of diversity, as is the case for the matrix/fusion non-coding region of the measles virus genome [36,37]. The rate of mutation per mumps gene was highest in the SH gene (Figure 6), as previously reported [38,39]. The SH gene consists of a small coding region, and most positions had a high variability. In contrast, other mumps genes did not show a high evolutionary rate.

### 3.4. Risk Factors for Mumps Infection

Epidemiological data was available for 82.3% (172/209) of the MB outbreak samples. Of these, 68.0% were aged 11–30 years, 52.9% were male, 72.1% lived rurally, and 73.3% were categorized as having low income. Using a logistic regression model (univariate analysis), the risk of acquiring mumps within this dataset in fully vaccinated individuals (those who had at least two doses of the MMR vaccine (*n* = 99)) was determined for the variables of age, sex, location (urban vs. rural), and income (low vs. high). In the univariate analysis, being male was shown to increase the risk of mumps infection by 1.84 times (*p* = 0.07), living in an urban setting had an increased risk of 1.67 (*p* = 0.22), and having a low-income level had an increased risk of 1.94 (*p* = 0.10). Age, which was a continuous variable in this analysis, was a significant risk factor for infection (OR 1.23, *p* < 0.01).

Exploring associations between non-synonymous mutations and epidemiological variables were also of interest. From the SNVPhyl pipeline, 16.7% (72/430) of SNVs were non-synonymous (Supplementary Table S2). There were no significant associations found between risk factors and specific SNVs. The risk of epidemiological factors in those infected with a mumps strain that contained a non-synonymous SNV vs. those that did not was then investigated. SNVs at positions 1813, 2996, 4828, and 8429 were excluded as they occurred in every MB sample. Living in an urban setting was associated with a reduced risk of having a mumps strain with a SNV (OR 0.26, *p* = 0.002). However, the majority of cases occurred in rural communities, introducing a bias [10]. Interestingly, the odds ratio of being infected with mumps virus with a mutation identified in this study was 8.0 (confidence interval (CI) 2.3–27.3) times higher for the 40 individuals who received only a single dose of MMR vaccine (*p* <0.001). While not achieving significance, the risk was 2.8 (CI- 0.7–11.3) times higher for the 85 individuals who received two doses (*p* = 0.14), and 4.5 (0.8 to 24.4) times higher for the 14 individuals who received three doses (*p* = 0.08). When the same analysis was run on synonymous mutations, no increase in the odds ratio was observed.

Of note, no multivariate analyses were conducted to assess potential confounding variables given the minimal data available for patient demographics.

## 4. Discussion

The analysis of MuV SH gene sequences in these outbreaks and in other similar settings has demonstrated that the SH gene sequences have little phylogenetic power in these settings. The SH gene sequencing lacked discrimination within the MB, NS, and NL outbreak, and individual chains of transmission or importations could not be resolved. Most samples had a single nucleotide difference (C248T) from the WHO reference strain for genotype G [40]. After exploration of the entropy of variants found through the genome, no alternate region to the SH gene was identified that showed a high level of diversity necessary for epidemiological investigations. This finding confirms that WGS is the best tool to provide greater amounts of phylogenetic information for mumps genotype G outbreak investigations [5,39,41] and in this study was shown that WGS clearly resolves the different Canadian outbreaks. Additionally, the use of WGS allows for confirmation of other mumps diagnostic tests such as real-time PCR. In this study, we mapped the mumps F gene diagnostic real-time primers to the WGS-t data set. One mutation was found in the probe and was present in 70.57% of samples. While this does not raise alarm on whether this assay will continue to work, the use of WGS allows for constant monitoring of mutations that may affect diagnostic tests in the future. Finally, the use of WGS allows for exploration of epitopes mismatches, which may give insight as to whether a genotype G-specific vaccine is required for mumps. Initial work on the effects of SNVs has been conducted within the laboratory.

In the Canadian mumps outbreaks, a Bayesian analysis was run on the final whole genome data, and the clock rate was determined to be 3.84 × 10^−4^ mutations/site/year (95% CI: 3.38 × 10^−4^, 4.29 × 10^−4^). This rate was similar to that found in an American mumps outbreak [6]. Given the stability that has previously been seen in the mumps SH gene, a high clock rate from the mutation data was not anticipated [42].

The inclusion of archival Canadian samples available from 2006 proved critical in this understanding of the epidemiology of genotype G evolution in Canada. Without the archival sequences, the tree would have suggested that the MB, NL, and NS outbreaks were due to different importations. However, WGS was able to distinguish these three outbreaks, with the MB outbreak being distinct from the outbreaks in NL and NS with a divergence in 2011, and a divergence of the NL and NS outbreaks in 2014 (Figure 4). Including the archival Canadian samples spanning multiple years and multiple provinces resulted in a tree shape compatible with the endemic transmission of mumps in Canada. Supporting endemic transmission, 70.4% of the MB outbreak cases were from the geographically isolated Northern Regional Health Authority [10]. The transmission of mumps cases within these communities was most likely not due to importation events but due to endemic virus circulation. All MB samples clustered together with divergence dates that fall between 2016–2017, which correlates with the timeline of the outbreak, again suggesting that there is endemic transmission of mumps in Canada. The distinct cluster between the three provincial outbreaks suggests that while mumps may be endemic in Canada, there is also local circulation within geographical areas and the possibility of importation events. The inclusion of BC WGS data showed what an importation event would look like if the provincial outbreaks had been due to multiple importations, as this different genetic SH profile cluster of BC samples separated from other Canadian samples on the tree but clustered with samples from India and New Zealand on a distant branch that has a divergence date of 1992. Altogether, these data demonstrate the utility of mumps genomic data for epidemiological investigations, furthering our understanding of transmission events within and between outbreaks.

A limitation of this study is that only three provincial outbreaks were included at this time. Most Canadian provinces have experienced mumps outbreaks [4]. To confirm the true endemic circulation of mumps within Canada, WGS of outbreak samples from other provinces would also need to be included. However, the current phylogenetic tree strongly suggests that even if more provinces were included, the data would still show endemic transmission, as archival sequences from provinces other than MB, NL, and NS still cluster within the more recent outbreak samples sequences.

Many countries have reported mumps outbreaks with similar characteristics to these outbreaks in Canada, they occur in young adults, in vaccinated populations, and are due to a genotype G virus [5,6,7,8,9]. In particular, the Netherlands and the US have reported and sequenced samples from outbreaks that mirror those seen in Canada [5,6,7]. For this reason, international sequences were included in the analysis.

The Netherlands and US samples clustered closely to the Canadian outbreaks. Samples from the US are found on the tree near both archival Canadian sequences, and within the more recent mumps outbreaks (Figure 4). This clustering suggests that mumps may not only be endemic in Canada but may be circulating in both Canada and the US. This would not be surprising as previous reports have documented the spread of mumps between these countries [43]. The sequences from the Netherlands and one sequence from Australia were also found within the Canadian samples. This could be due to travel-related spread of mumps.

The Bayesian analysis strongly suggests that mumps is endemically circulating in Canada. Endemic transmission of the mumps virus in a highly immunized population suggests that the current vaccine, vaccination rate and surveillance methods are not adequate to reach elimination status. The current Canadian target for mumps is to have less than 100 cases per year over a 5-year rolling average, but current outbreak numbers are much higher than this [44]. As many of the cases are occurring in vaccinated individuals, this goal will not be achieved without changes in vaccination and surveillance policies. The national vaccination goal for mumps is to reach 95% coverage by the age of seven years [44]. The 2017 Childhood National Immunization Coverage Survey indicated only 90% of children had received one dose of the MMR vaccine by the age of two, and 86% had received both doses required for a full MMR immunization by the age of seven [3]. Additionally, mumps was already the least effective component of the MMR vaccine, and with cases being almost exclusively genotype G while the vaccine is genotype A, a mismatch in antibody production may be contributing to these outbreaks [45]. The current global vaccination program is not sufficient to achieve elimination status.

Mapping of the nucleotide variant profile of samples indicated some variants were shared between provinces, while others were found only in one province or a geographical area. For example, the SNV A134G was found both in archival sequences and the NL/NS sequences, SNVs C1860T, T4447G, C4501T, C4504T, G5428A, and C6020T were mainly found in archival samples and were present in only a small number of outbreak samples. This indicates that these variants have been lost or selected against over time or that these are independent evolutions of clades from a common ancestor. As both similarities and differences were observed in the variants in the provincial outbreaks, one could hypothesize that there are strains circulating locally within provinces/regions of Canada. It should be noted that this study is limited by the number of outbreaks that were sequenced, as many other outbreaks were reported during a similar time frame in Canada [4]. To properly understand if variant profiles are specific within regions of Canada, other provincial outbreaks would need to be sequenced and included in the analysis.

With whole genome data and some patient demographics available, the large MB outbreak provided an excellent opportunity to perform a preliminary exploration of possible risk factors for mumps infection. Using a logistical regression model, many variables were compared, including age, sex, income status, and setting (urban or rural); however, these analyses could be skewed by confounding variables including those not captured in the data collection. A more robust analysis using multivariate regression models would have been more appropriate for these investigations. Nonetheless, there was an interesting finding from the comparison of non-synonymous mutations occurring between non-vaccinated and vaccinated individuals. The risk of being infected with a mumps strain that had a non-synonymous SNV was increased significantly by a factor of eight in those with only one dose of an MMR vaccine. While it could be speculated that those with only one MMR dose could be driving mutation rates within an outbreak and could be a primary target group for public health intervention in future outbreaks, it should also be noted that wide confidence intervals were observed. There seemed to be a similar but non-significant increase in the risk of having a non-synonymous SNVs in individuals having received two and three MMR vaccines as well, but again wide confidence intervals prevented conclusive results. Further investigations should try to increase specimen numbers to determine if these observations hold true.

## 5. Conclusions

Overall, the key findings of this study include the Bayesian analysis of the MB, NL, and NS outbreaks and archival Canadian sequences that showed the Canadian mumps outbreaks are compatible with endemic transmission and not exclusively from imported cases. The confirmation of endemic transmission of mumps indicates that, unlike measles and rubella, circulation of mumps has not been interrupted in Canada and an increase in public health measures and surveillance will be required to reach elimination status. WGS-based surveillance has shown its merits compared to traditional Sanger sequencing of the SH gene and should be the tool of choice for genomics mumps investigation in highly vaccinated populations. Future work in studying outbreaks that cover a larger area of Canada, as well as the relationship between Canadian and American samples will allow for more in-depth analysis of mumps virus circulation. Understanding the genetic evolution of mumps genotype G, as well as identifying mutations leading to vaccine escape would be critical information for novel mumps vaccine development and might provide the key to mumps virus elimination. Ongoing and increased molecular surveillance and research on alternative vaccination strategies for mumps should be encouraged.

## Figures and Tables

**Table 1:** Distribution of income quartiles used for this study.

Quartiles	Income	Rural (CAD)	Urban (CAD)
1	Low income	28,596.00–59,575.00	20,437.00–59,967.00
2		59,612.25–72,376.67	59,972.00–75,575.50
3	High Income	72,412.50–81,348.33	75,585.00–92,708.00
4		81,488.71–95,903.43	92,709.00–116,183.00
5		95,941.00–264,050.00	116,228.00–689,245.00

## Data Availability

The sequences generated in this study are available on GenBank under the accession numbers PQ156981 - PQ157298.

## Supplementary Materials

The following supporting information can be downloaded at: https://www.mdpi.com/article/10.3390/v16081280/s1, Table S1: Results from the BEAST model testing; Table S2: Properties of the significant single nucleotide variants found from whole genome sequencing data from the Manitoba, Newfoundland, and Nova Scotia outbreaks; Figure S1: Maximum Likelihood tree of the SH sequences obtained through Sanger sequencing of Manitoba samples (pink), Newfoundland samples (green) and Nova Scotia samples (Blue); Figure S2: Likelihood mapping of MuV Sanger SH gene sequences from the Manitoba, Newfoundland, and Nova Scotia outbreaks.
