# Supplementary material for: Genomic Characterization of Three Canadian Mumps Outbreaks Demonstrates Endemic Transmission in Canada
Source: Viruses. 2024 Aug 10;16(8):1280. doi: 10.3390/v16081280 (PMC11359846; doi:10.3390/v16081280)
Supplement: Supplementary file 1 [file viruses-16-01280-s001.zip › viruses-3114187-supplementary.pdf]

Supplementary Info

Tables:

Table S1: Results from the BEAST model testing. Models were run in triplicate with 50,000,000 chains sampled at every 5000 trees. Strict and Relaxed models are represented by the marginal likelihood predicted through path sampling in BEAST 2 (shown as negative values) on average by each model. The clock rate for models ran with the strict clock (mutation rate/site/year) are shown with the 95% confidence intervals (CI) in brackets.

Model	Strict Clock		Relaxed Clock
	Marginal likelihood	Clock rate (95%CI)	Marginal likelihood
Coalescent Constant Population	-39993.71	3.85×10^{-4} (3.41×10^{-4}- 4.32×10^{-4})	-48841.82
Coalescent Exponential Population	-39963.15	3.83×10^{-4} (3.47×10^{-4}- 4.26×10^{-4})	-48808.20
Coalescent Bayesian Skyline	-45601.82	3.77×10^{-4} (3.34×10^{-4}- 4.24×10^{-4})	-48800.82

Table S2: Properties of the significant single nucleotide variants found from whole genome sequencing data from the Manitoba, Newfoundland, and Nova Scotia outbreaks. ‘Position’ refers to the nucleotide at which the change occurs. ‘Archival samples’ are those that were sampled from 2006-2017, the samples classified as ‘Other Canadian’ include those in the outlier BC outbreak and genotype G laboratory strains. Under the ‘status’ column, valid refers to mutations that passed the minimum sequencing depth and quality requirements in the SNVPhyl pipeline, while filtered-coverage refers to those that missed the sequencing depth requirement in 1-5 samples.

AA Change	Status	Reference	Position	SNV	# of Samples	Manitoba	Newfound-land	Nova Scotia	Archival Canadian	Other Canadian
**Q to R**	Valid	A	116	G	10	1				9
**L to F**	Valid	C	127	T	10	1				9
**H to R**	Valid	A	134	G	67		21	45	6	1
**Q to H**	Valid	A	1513	G	39			39		
**K to N**	Valid	A	1813	T	246	203		39		4
**S to N**	Valid	G	1848	A	23	23				
**P to L**	Valid	C	1860	T	12	2			11	10
**S to F**	Valid	C	1869	T	7	7				
**L to S**	Valid	T	2079	C	10	1				9
**K to R**	Valid	A	2208	G	10	1				9
**Q to K**	Valid	C	2312	A	39			39		
**G to R**	Valid	G	2996	A	207	204				3
**D to E**	Valid	T	3314	A	39			39		
**S to L**	Valid	C	3493	T	7	7				
**V to I**	Valid	G	3882	A	39			39		
**S to P**	Valid	C	4151	T	8		7	1		
**S to P**	Valid	T	4419	C	11	1			4	10
**L to ***	Valid	T	4447	G	13	2		1	23	10
**I to V**	Valid	A	4455	G	10	1				9
**P to L**	Valid	C	4501	T	12	2			12	10
**P to L**	Valid	C	4504	T	12	2			12	10
**P to S**	Valid	C	4828	T	207	204				3
**S to N**	Valid	G	5438	A	13	2		1	21	10
**P to S**	Valid	G	5949	T	27	27				
**A to T**	Valid	G	6019	A	5		4	1		
**A to V**	Valid	C	6020	T	13	2		1	23	10
**M to I**	Valid	G	6039	T	8	1				7
**I to R**	Valid	T	6197	G	10	1				9
**T to A**	Valid	A	6334	G	5	5				
**I to T**	filtered-coverage	T	6350	C	21	21				
**I to T**	filtered-coverage	T	6353	C	21	21				
**V to I**	Valid	G	6361	A	11	1		8		2
**A to S**	Valid	G	6376	T	8	8				
**H to R**	Valid	A	6416	C	39			39		
*** to Q**	Valid	T	6559	C	5	3				2
**S to I**	Valid	G	6675	T	9	1				8
**N to D**	Valid	A	6686	G	8	1				7

**A to V**	Valid	C	6723	T	9	1				8
**T to I**	Valid	C	6744	T	28			28		
**V to I**	Valid	G	7358	A	31	29				2
**T to A**	Valid	A	8072	G	22		20	2		
**S to G**	filtered-coverage	A	8384	G	10	1				9
**S to L**	Valid	C	8412	T	30	30				
**Q to ***	filtered-coverage	C	8429	T	84	79	1	1		3
*** to W**	Valid	A	15243	G	13	2		1		10
**S to P**	Valid	T	15278	C	70	1	21	46		2
**L to F**	Valid	C	15287	T	11	2				9
**Q to***	filtered-coverage	C	15317	T	10	1				9

Figures:

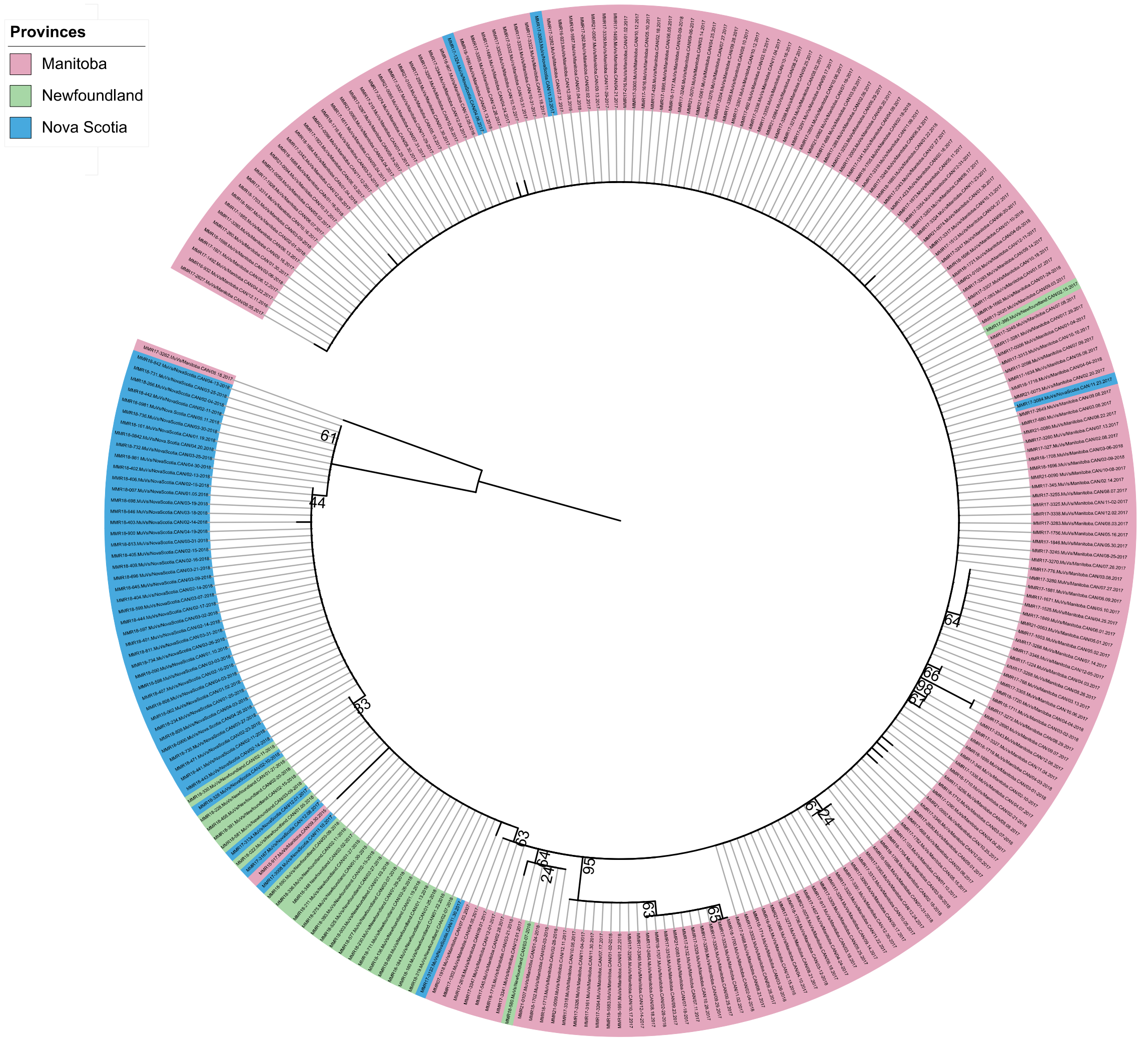

Figure S1: Maximum Likelihood tree of the SH sequences obtained through Sanger sequencing of Manitoba samples (pink), Newfoundland samples (green) and Nova Scotia samples (Blue). A bootstrap analysis of 1000 was run, percentages are indicated at the nodes (those less than 10 are not shown on the tree). The tree can also be viewed at https://itol.embl.de/shared/18FQx6JYPbiX5.

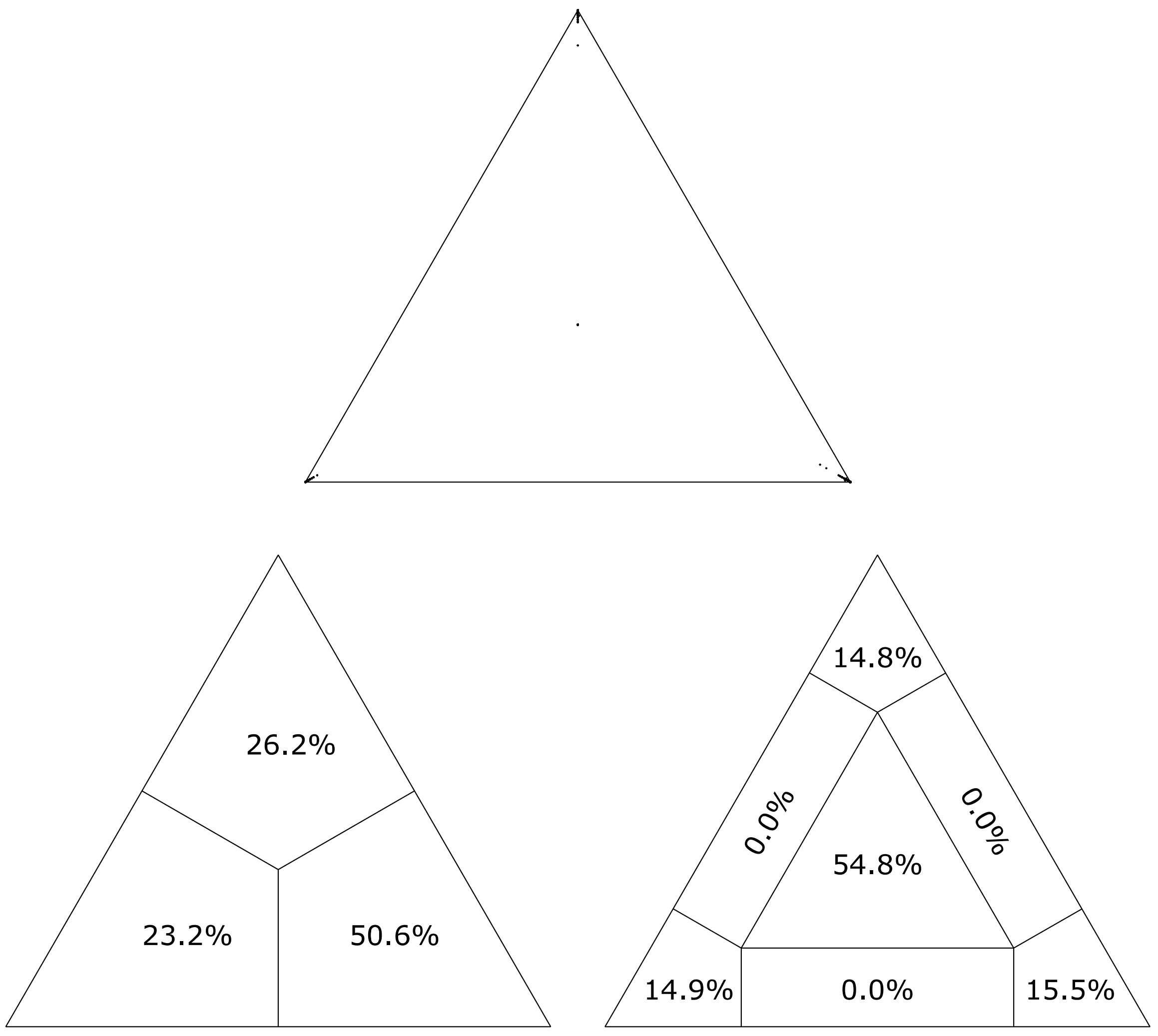

Figure S2: Likelihood mapping of MuV Sanger SH gene sequences from the Manitoba, Newfoundland, and Nova Scotia outbreaks. IQ-TREE 2.1.3 was run with 5000 quartets indicated. The top triangle indicates the distribution of quartets. The left triangle indicates the percentage of quartets falling into one of the 3divided areas. The bottom right triangle shows how the samples were placed, with 45.2% being placed at the corners, indicating weak phylogenetic power.
